# A three-week in-hospital multidisciplinary body weight reduction program exerts beneficial effects on physical and mental health and fatiguability of elderly patients with obesity

**DOI:** 10.3389/fnagi.2022.1054941

**Published:** 2022-12-16

**Authors:** Anna Guerrini Usubini, Michela Bottacchi, Adele Bondesan, Diana Caroli, Gianluca Castelnuovo, Alessandro Sartorio

**Affiliations:** ^1^Department of Psychology, Catholic University of Milan, Milan, Italy; ^2^Istituto Auxologico Italiano IRCCS, Psychology Research Laboratory, Milan, Italy; ^3^Istituto Auxologico Italiano, Istituto di Ricovero e Cura a Carattere Scientifico (IRCCS), Experimental Laboratory for Auxo-Endocrinological Research, Piancavallo-Verbania, Italy; ^4^Istituto Auxologico Italiano, Istituto di Ricovero e Cura a Carattere Scientifico (IRCCS), Experimental Laboratory for Auxo-Endocrinological Research, Milan, Italy

**Keywords:** body weight reduction program, obesity, elderly patients, fatigue, psychological well-being

## Abstract

**Introduction:**

Obesity represents one of the most serious problems of public health affecting elderly populations in an increasingly relevant way. The aim of the current study was to assess the effects of a 3-week in-hospital multidisciplinary body weight reduction program (BWRP) in a sample of elderly patients with obesity on reducing body mass index (BMI), improving fatigue, muscle performance, and psychological well-being.

**Methods:**

Two hundred and thirty-seven consecutive elderly in-patients with obesity (males = 84; females = 153; age range = 65–86 yrs.; mean BMI = 43.7) undergoing a three-week multidisciplinary BWRP participated in the study. Data on BMI, fatiguability (measured with the Fatigue Severity Scale, FSS), muscle performance (evaluated with the Stair Climbing Test, SCT), and psychological well-being (assessed with the Psychological General Well- Being Index, PGWBI) were collected before and after the intervention.

**Results:**

Results showed that BWRP was capable to reduce BMI [*F*(1.00, 235.00) = 1226.8; *p* < 0.001; *ƞ*^2^ = 0.024], improve perceived fatigue [*F*(1,234) = 296.80125; *p* < 0.001; *ƞ*^2^ = 0.129], physical performance [*F*(1.00,158.00) = 119.26; *p* < 0.001; *ƞ*^2^ = 0.026], and enhance psychological well-being [*F*(1,235) = 169.0; *p* < 0.001; *ƞ*^2^ = 0.103] in both males and females.

**Discussion:**

Although it will be necessary to demonstrate with further longitudinal studies whether the reported beneficial effects will be maintained over time, the effectiveness of a 3-week BWRP on different aspects involved in determining a level of autonomy and good quality of life of elderly obese patients appears to represent a valid attempt to counteract – at least in part – the unavoidable and progressive disability of these patients.

## 1. Introduction

Obesity represents one of the most serious problems of public health affecting over 600 million adults worldwide (Gutiérrez-Fisac and Rodríguez-Artalejo, 2006; World Health Organization, 2006; Davin and Taylor, 2009; Ayensa and Calderon, 2011; Castelnuovo et al., 2015; DerSarkissian et al., 2017). The World Health Organization European Regional Obesity Report 2022 (World Health Organization, 2022) pointed out that 59% of European adults are living with overweight or obesity. In addition, more than one-fifth of adults in 49 out of 53 member states are living with obesity, with levels reaching one-third in several countries. While overweight is higher among males (63%) than females (54%) across the European countries, on the contrary, obesity is more prevalent in females (24%) than males (22%) in about half of the European countries. Importantly, the prevalence of overweight and obesity is still rising with justified concerns about the impact on public health and economies.

Obesity is associated with many medical conditions, such as s type 2 diabetes, cardiovascular diseases, chronic back pain, obstructive sleep apnoea syndrome, obesity hypoventilation syndrome, gallbladder disease, liver diseases, gout, and several common forms of cancer. In addition, individuals with obesity are at risk for many mental conditions, such as depression and anxiety disorders (Wadden et al., 2002; Byrne et al., 2004; Klein et al., 2004; Flegal et al., 2005; Dong et al., 2006; Castelnuovo et al., 2014; Boles et al., 2017).

The condition of obesity is strongly related to many forms of disability. Excess of adiposity negatively influences postural control and reduces functional mobility, causing impairment in stability and walking speed. In addition, obesity is often associated with a higher perception of fatigue, a subjective difficulty to carry out voluntary activities often accompanied by a lack of energy, apathy, and tiredness. As far as psychological health is concerned, many individuals with obesity are more likely to deal with psychological issues, such as depression, anxiety, low self-esteem, and reduced quality of life. Several studies found that people suffering from obesity were almost five times more likely to have experienced an episode of major depression in the past years, as compared with their healthy counterparts (Onyike et al., 2003). In addition, the relationship between obesity and depression seems to be stronger for women than men (Carpenter et al., 2000). People with obesity are also more likely to suffer from anxiety disorders, specifically social anxiety (Kalarchian et al., 2007; Sarwer et al., 2012), and their quality of life is generally impaired. Frequently, individuals with obesity are stigmatized and discriminated, with negative effects on their general life functioning (Friedman et al., 2008).

Obesity is a complex chronic disease, with multifaceted causes and several health consequences. This means that no single intervention alone can flat the constant rise of the obesity epidemic, but rehabilitation programs are required to address the clinical needs of individuals with obesity. Existing clinical guidelines for the treatment and management of obesity recommend comprehensive multidisciplinary and multiprofessional lifestyle interventions for weight loss. Such interventions should consist of nutritional, physical, and psychological components (Giusti et al., 2020).

The potential benefits associated with weight loss in patients with obesity should be not only established but also constantly updated. While the short and long-term effects of BWRP on adult and pediatric populations have been well-established by previous studies performed by our group (Lazzer et al., 2020; Rigamonti et al., 2020a,b) little attention has been paid on to elderly obese people so far (Flegal et al., 2016; Rigamonti et al., 2019).

The aim of the current study, therefore, was to assess the effects of a 3-week in-hospital multidisciplinary body weight reduction program (BWRP) consisting of diet, physical activity, psychological support and nutritional counseling in a sample of elderly people with obesity. Outcomes were BMI, fatigue, muscle performance, and psychological well-being in both sexes, which were compared. Our hypothesis was that BWRP produced improvements in BMI, fatigue, muscle performance, and psychological well-being.

## 2. Materials and methods

### 2.1. Participants and procedures

Two hundred and thirty-seven consecutive elderly inpatients with obesity (mean BMI: Kg/m^2^: 43.7) aged between 65 and 86 yrs., without any physical or psychological condition that could compromise their participation in the study were recruited for the study. Participants were included if they were Italian, older than 64 yrs., with a BMI greater than 35 (World Health Organization, 2006). Exclusion criteria included any form of physical or psychological impairment that could have compromised the participation in the study. Participants were referred to the Division of Metabolic Diseases, IRCCS Istituto Auxologico Italiano, Piancavallo-Verbania, Italy, a specialized clinical center (i.e., third level) offering a 3-week in-hospital multidisciplinary BWRP. At the admission to the hospital, patients who met the inclusion/exclusion criteria were informed about the study and were asked to provide written informed consent to participate.

Before (baseline, T0) and after 3-week BWRP (post BWRP, T1), participants were asked to fill in a battery of self-report questionnaires used to assess the variables of interest for the study. To calculate BMI weight and height were assessed by the medical team.

All the variables have been measured in the total sample (237), except for the SCT (muscle performance) which has been assessed only in those who had the necessary physical capabilities to perform this test (160). For this reason, 77 patients were excluded for their inability to do SCT. The eventual differences between subgroups (physically healthy to do SCT versus. physically impaired to do SCT) were out of interest for this paper. This is the reason why we analyzed all the other variables in the total sample (237).

The study was approved by the Ethical Committee of Istituto Auxologico Italiano (registration code: 2013_06_27; project code: 18A301) and followed the Helsinki Declaration and its later advancements.

#### 2.1.1. Body weight reduction program

The BWRP lasted 3 weeks. During this period, patients were placed on a hypocaloric diet. The amount of energy to be given with diet was calculated by subtracting approximately 25% from the total energy expenditure, which is obtained by multiplying the estimated daily resting energy expenditure (eREE) by the physical activity level during the BWRP, as previously described (Tamini et al., 2021). REE was estimated using the Mifflin equation (Mifflin, 1990) as follows: eREE = 9.99 × weight (kg) + 6.25 × height (cm) − 4.92 × age (years) + 166 × sex – 161, where sex values are = 1 for males and = 0 for females. In terms of macronutrients, the diet contained 21% proteins, 53% carbohydrates, and 26% lipids. The diet composition was formulated according to the Italian recommended daily allowance (ISo Nutrition, 1996). Each patient was free to choose foods from a heterogeneous daily menu, although five daily servings of fruits and vegetables were mandatory. Foods to which the patient reported allergic reactions were eliminated from the menu. A fluid intake of at least 1.5 l/day was encouraged. In addition, the dietitian team checked that each subject had eaten every meal. On each day of the BWRP, the patients had dietetics classes consisting of lectures, demonstrations, and group discussions with and without a supervisor. They also followed a daily program of adapted physical activity at moderate intensity (1 h) with indoor light jogging, dynamic exercises of the upper and lower limbs, 20–30 min of aerobic activity, postural gymnastics, and walking (3–4 km). Finally, they received psychological counseling based on Cognitive Behavioral Therapy with individual and group sessions provided once a week. The psychological intervention is aimed at helping patients to develop coping strategies and problem-solving skills, enhance self-efficacy, improve stimulus control and foster social activation measures (Giusti et al., 2020).

### 2.2. Measures

#### 2.2.1. Body mass index

Body Mass Index (BMI) was calculated by dividing the person’s weight in kilograms by the height in meters squared according to the proper formula: BMI = (kg/m^2^).

#### 2.2.2. Fatigue

The Fatigue Severity Scale (FSS) (Hjollund et al., 2007; Impellizzeri et al., 2013) is one of the most commonly used self-report questionnaires to assess the impact of fatigue on motivation, exercise, physical functioning, carrying out duties, interfering with work, family, or social life. The FSS consists of 9 statements about the impact of fatigue on functioning (e.g., “My motivation is lower when I am fatigued”) each rated on a scale from 1 “strongly disagree” to 7 “strongly agree.” The total score is obtained by the mean of item scores.

#### 2.2.3. Psychological well-being

The Psychological General Well-Being Index (PGWBI) (Dupuy, 1984; Grossi et al., 2006) is a well-validated questionnaire used in different contexts, including clinical trials and research to assess health-related quality of life (Grossi et al., 2006). It is composed of 22 items rated on a 6-point Likert scale which address six dimensions: anxiety (PGWBI-A), depression (PGWBI-D), positive well-being (PGWBI-PWB), self-control (PGWBI-SC), general health (PGWBI-GH), and vitality (PGWBI-V). The sub-scales scores and the total score (PGWBI-TOT) are obtained by the sum of item scores. Higher total scores indicated greater well-being. Higher scores in Anxiety and Depression subscales indicated less anxiety and depression, while lower scores in those subscales suggest greater anxiety and depression.

#### 2.2.4. Muscle performance

The Stair climbing test (SCT) (Margaria et al., 1966; Sartorio et al., 2001; Lafortuna et al., 2002) was used to measure the maximal anaerobic power muscles. Participants were asked to climb up ordinary stairs (13 steps of 15.3 cm each, with a total vertical distance of 1.99 m) at the highest possible speed, according to their capabilities. An experimenter measured the time employed to cover the test with a digital stopwatch. In line with Margaria assumptions, anaerobic power (in W) was calculated by using the following formula: [(Kg × 9:81 × 1.99) /s] where kg was body mass, 9.81 m/s^2^ was the acceleration of gravity, 1.99 m was vertical distance and s was time.

### 2.3. Statistical analysis

Descriptive statistics were computed to assess the baseline characteristics of the patients participating in the study. To assess changes from pre-to-post intervention in all the study variables, several mixed between within 2 (groups: males versus. females) × 2 (times: baseline versus. post BWRP) repeated measures analysis of variance (ANOVAs) were conducted. Effect size (*η*^2^) was used to quantify the global difference of the two groups across times and was interpreted with the following benchmarks: null (*η*^2^ < 0.003); small (0.003 < *η*^2^ > 0.003 to 0.039); moderate (0.110 < *η*^2^ > 0.40); and large (*η*^2^ > 0.110; Cohen, 2013). In addition, Spearman’s rho correlation between pre–post BWRP difference (∆ %) of FSS (total score) and SCT time (s) have been assessed.

Analyzes were performed using Jamovi (The jamovi project 2021). Jamovi (Version 1.6) [Computer Software] retrieved from https://www.jamovi.org.

## 3. Results

The baseline characteristics of the sample are shown in Table 1. The sample was composed of 153 (64.6%) females and 84 (35.4%) males. The mean age of males was 69.9 (SD = 4.11) and the mean age of females was 71 (SD = 4.46), these values being not significantly different. By exploring pre-test conditions, females had a higher BMI (*p* < 0.001), greater levels of FSS (*p* = 0.015), worse SCT (*p* < 0.001), and lower levels of PGWBI-TOT (*p* < 0.001) than males of comparable age.

**Table 1:** Descriptive characteristics of the study group.

	Males (*N* = 84)			Females (*N* = 153)		
Variables	T0	T1	Value of *p*	T0	T1	Value of *p*
BMI	42.2 ± (4.05)	40.03 ± (3.91)	<0.001	44.6 ± (5.18)	43.02 ± (5.05)	<0.001
∆ BMI	−1,77 ± (0.74)			−1.46 ± (0.63)		
FSS	39.06 ± (13.5)	29.9 ± (11.5)	<0.001	43.8 ± (12.5)	34.2 ± (10.4)	<0.001
∆ FSS	−9.69 ± (8.71)			−9.61 ± (7.97)		
PGWBI-A	17.9 ± (4.82)	20.5 ± (2.08)	<0.001	15.5 ± (5.17)	19.8 ± (2.37)	<0.001
PGWBI-D	12.0 ± (2.91)	13.0 ± (2.27)	<0.001	10.6 ± (3.26)	12.05 ± (2.50)	<0.001
PGWBI-PWB	11.8 ± (3.84)	13.8 ± (3.43)	<0.001	9.19 ± (3.90)	12.3 ± (3.63)	<0.001
PGWBI-SC	11.4 ± (3.10)	12.5 ± (2.80)	<0.001	9.86 ± (3.28)	11.3 ± (2.97)	<0.001
PGWBI-GH	9.23 ± (3.13)	9.81 ± (2.74)	<0.001	7.29 ± (2.84)	8.73 ± (2.82)	<0.001
PGWBI-V	11.6 ± (4.23)	13.4 ± (3.50)	<0.001	9.29 ± (3.92)	12.4 ± (3.82)	<0.001
PGWBI-TOT	74.1 ± (18.4)	83.0 ± (13.5)	<0.001	61.7 ± (18.9)	77.0 ± (14.0)	<0.001
	Males (*N* = 64)			Females (*N* = 96)		
	T0	T1		T0	T1	
SCT	5.88 ± (1.82)	5.25 ± (1.66)	<0.001	8.64 ± (2.83)	7.56 ± (2.26)	<0.001
∆ SCT	−0.625 ± (0.557)			−1.08 ± (1.16)		

T0, baseline; T1, after 3-week BWRP; BMI, Body Mass Index; FSS, Fatigue Severity Scale; SCT, Stair Climbing Test; PGWBI-A, Psychological General Well-Being Index-Anxiety; PGWBI-D, Psychological General Well-Being Index-Depression; PGWBI-PWB, Psychological General Well-Being Index-Positive Well-Being; PGWBI-SC, Psychological General Well-Being Index-Self-Control; PGWBI-GH, Psychological General Well-Being Index-General Health; PGWBI-V, Psychological General Well-Being Index-Vitality; PGWBI-TOT, Psychological General Well-Being Index-Total Score. Data are expressed as means and standard deviations.

### 3.1. Effects of BWRP on BMI

Results showed a significant main effect of time suggesting that BMI significantly decreased from baseline to post BWRP [*F*(1.00, 235.00) = 1226.8; *p* < 0.001; *ƞ*^2^ = 0.024] independently from sex, and a significant main effect of group [*F*(1,235) = 18.0; *p* < 0.001; *ƞ*^2^ = 0.069]. This means that there was a significant difference in the BWRP-induced effects on BMI between males and females. The interaction effect of time x group was significant [*F*(1.00, 235.00) = 10.9; *p* = 0.001; *ƞ*^2^ = 0.000], indicating that the change from baseline to post BWRP was different depending upon the two groups (males versus. females). In particular, independent sample t-tests showed that males reported lower BMI than females both at baseline and post BWRP (*p* < 0.001 respectively). However, the two groups decreased significantly BMI from baseline to post BWRP (*p* < 0.001 respectively).

### 3.2. Effects of BWRP on fatigue

Results showed that fatigue significantly decreased from baseline to post BWRP [*F*(1,234) = 296.80125; *p* < 0.001; *ƞ*^2^ = 0.129] independently from sex. In addition, there was a significant difference in the BWRP-induced effects on fatigue between males and females [F(1,234) = 8.07; *p* = 0.005; *ƞ*^2^ = 0.026]. The interaction effect of time x group was not significant [*F*(1, 234) = 0.00493; *p* = 0.944; *ƞ*^2^ = 0.000].

### 3.3. Effects of BWRP on psychological well-being

Results showed that psychological general well-being (PGWBI-TOT) significantly decreased from baseline to post BWRP [*F*(1,235) = 169.0; *p* < 0.001; *ƞ*^2^ = 0.103] independently from sex. In addition, there was a significant difference in the BWRP-induced effects on PGWBI-TOT between males and females [*F*(1,235) = 20.04; *p* < 0.001; *ƞ*^2^ = 0.060]. The interaction effect of time x group was not significant [*F*(1,235) = 11.9; *p* < 0.001; *ƞ*^2^ = 0.060].

As far as anxiety (PGWBI-A) is concerned, results showed that anxiety (PGWBI-A) significantly decreased from baseline to post BWRP [*F*(1,235) = 104.42; *p* < 0.001; *ƞ*^2^ = 0.145] independently from sex. In addition, we found a significant main effect of group [*F*(1,235) = 14.8.; *p* < 0.001; *ƞ*^2^ = 0.060] suggesting that there was a significant difference in the BWRP-induced effects on PGWBI-A between males and females. The interaction effect of time x group was significant [*F*(1,235) = 6.42; *p* = 0.012; *ƞ*^2^ = 0.009], indicating that the change from baseline to post BWRP was different depending upon the two groups (males versus. females). In particular, independent sample t-tests showed that males reported lower PGWBI-A than females both at baseline (*p* < 0.001), and post BWRP (*p* = 0.018). However, the two groups decreased significantly in PGWBI-A from baseline to post BWRP (*p* < 0.001 respectively).

Results pointed out that depression (PGWBI-D) significantly decreased from baseline to post BWRP [*F*(1,235) = 68.69; *p* < 0.001; *ƞ*^2^ = 0.055] independently from sex. In addition, there was a significant difference in the BWRP-induced effects on PGWBI-D between males and females [*F*(1,235) = 8.30; *p* = 0.004; *ƞ*^2^ = 0.026]. The interaction effect of time x group was significant [*F*(1,235) = 6.60; *p* = 0.011; *ƞ*^2^ = 0.005], indicating that the change from baseline to post BWRP was different depending upon the two groups (males versus. females). In particular, independent sample t-tests showed that males reported lower PGWBI-D than females at baseline (*p* < 0.001), while at post BWRP the difference was not significant (*p* = 0.106). However, the two groups decreased significantly in PGWBI-D from baseline to post BWRP (*p* < 0.001 respectively).

As far as positive well-being (PGWBI-PWB) is concerned, results showed a significant main effect of time suggesting that positive well-being (PGWBI-PWB) significantly increased from baseline to post BWRP [*F*(1,235) = 151.82; *p* < 0.001; *ƞ*^2^ = 0.093] independently from sex, and a significant main effect of group [*F*(1,235) = 20.0; *p* < 0.001; *ƞ*^2^ = 0.059]. This means that there was a significant difference in the BWRP-induced effects on PGWBI-PWB between males and females. The interaction effect of time x group was significant [*F*(1,235) = 6.39; *p* = 0.012; *ƞ*^2^ = 0.004], indicating that the change from baseline to post BWRP was different depending upon the two groups (males versus. females). In particular, independent sample t-tests showed that males reported higher PGWBI-PWB than females both at baseline (*p* < 0.001) and post BWRP (*p* = 0.002). However, the two groups improved significantly in PGWBI-PWB from baseline to post BWRP (*p* < 0.001 respectively).

Self-control (PGWBI-SC) significantly increased from baseline to post BWRP [*F*(1,235) = 48.34; *p* < 0.001; *ƞ*^2^ = 0.036] independently from sex. In addition, we found a significant difference in the BWRP-induced effects on PGWBI-SC between males and females [*F*(1,235) = 13.0; *p* < 0.001; *ƞ*^2^ = 0.041]. The interaction effect of time x group was not significant [*F*(1,235) = 1.66; *p* = 0.198; *ƞ*^2^ = 0.001].

Results about general health (PGWBI-GH) showed a significant main effect of time suggesting that PGWBI-GH significantly increased from baseline to post BWRP [*F*(1,235) = 33.20; *p* < 0.001; *ƞ*^2^ = 0.026] independently from sex. In addition, there was a significant difference in the BWRP-induced effects on PGWBI-GH between males and females [*F*(1,235) = 18.7; *p* < 0.001; *ƞ*^2^ = 0.058]. The interaction effect of time x group was significant [*F*(1,235) = 5.94; *p* = 0.016; *ƞ*^2^ = 0.005], indicating that the change from baseline to post BWRP was different depending upon the two groups (males versus. females). In particular, independent sample t-tests showed that males reported higher PGWBI-GH than females both at pre-test (*p* < 0.001) and post-test (*p* = 0.005). However, the two groups improved significantly in PGWBI-GH from baseline to post BWRP (*p* < 0.001 respectively).

Finally, vitality (PGWBI-V) significantly increased from baseline to post BWRP [*F*(1,235) = 116.25; *p* < 0.001; *ƞ*^2^ = 0.081] independently from sex. In addition, we found a significant main effect of group [*F*(1,235) = 12.3; *p* < 0.001; *ƞ*^2^ = 0.037] suggesting that that there was a significant difference in the BWRP-induced effects on PGWBI-V between males and females. The interaction effect of time x group was significant [*F*(1,235) = 8.07; *p* = 0.005; *ƞ*^2^ = 0.006], indicating that the change from baseline to post BWRP was different depending upon the two groups (males versus. females). In particular, independent sample t-tests showed that males reported higher PGWBI-V than females both at baseline (*p* < 0.001) and post BWRP (*p* = 0.045). However, the two groups improved significantly in PGWBI-V from baseline to post BWRP (*p* < 0.001 respectively).

### 3.4. Effects of BWRP on muscle performance

Among participants, a subgroup of 160 patients were assessed for their physical capability to go up a flight of stairs, a simple, repeatable, and safe test used to evaluate lower limb muscle capacity (by SCT) before and after the BWRP, according to their own capabilities, in order to assess changes from pre-to-post intervention. Seventy-seven patients were excluded to perform this test due to their physical inability at the admission to the hospital.

Results showed a significant main effect of time suggesting that time requested to perform SCT significantly reduced from baseline to post BWRP [*F*(1.00,158.00) = 119.26; *p* < 0.001; *ƞ*^2^ = 0.026] independently from sex, and a significant main effect of group [*F*(1,158) = 50.0; *p* < 0.001; *ƞ*2 = 0.226]. This means that there was a significant difference in the BWRP-induced effects on SCT between males and females. The interaction effect of time x group was significant [*F*(1.00, 158.00) = 8.47; *p* = 0.004; *ƞ*^2^ = 0.002], indicating that the change from baseline to post BWRP was different depending upon the two groups (males versus. females). In particular, independent sample t-tests showed that males reported better SCT (time) than females both at baseline and post BWRP (*p* < 0.001 respectively). However, the two groups improved significantly in SCT (time) from baseline to post BWRP (*p* < 0.001 respectively).

### 3.5. Interaction between fatigue and muscle performance

Analyzing data from all participants who were tested with SCT, we assessed the relationship between the BWRP-induced effects on fatigue (FSS) and SCT time, hypothesizing that the reduction in fatigue due to the BWRP was correlated to an improvement of SCT (i.e., less time to climb the stair). No correlation was found between pre–post BWRP difference (∆ %) of FSS (total score) and SCT time (s) (*ρ* = −0.016; *p* = 0.804). See Supplementary Figure S1.

## 4. Discussion

According to the main findings of the present study, 3-week in-hospital multidisciplinary BWRP was capable to reduce BMI, reduce fatigue (FSS), improve muscle performances (SCT), and enhance psychological well-being (PGWBI) in a sample of elderly people with obesity. In particular, anxiety and depression (PGWBI-A and PGWBI-D) significantly decreased, while positive well-being, self-control, general health, and vitality (PGWBI-PWB, PGWBI-SC; PGWBI-GH, and PGWBI-V respectively) increased. These results were achieved both in males and females with positive effects in all the study variables in both genders, even if the baseline and post-intervention conditions of females were worse than those of male participants (i.e., greater BMI, higher FSS, lower SCT, and lower PGWBI). According to our results, at the end of the intervention males reported greater psychological conditions than females as indicated by lower levels of PGWBI-A and PGWBI-D, as well as greater rates of PGWBI-PWB, PGWBI-GH, and PGWBI-V.

The better starting condition of elderly males was associated with a better final condition than in females, thus indicating that the positive effects exerted by 3-week BWRP were obtained irrespective of the baseline conditions (greater or lower general impairment).

The baseline and final worse psychological conditions of females, as compared to males are not to be considered surprising, since the psychological conditions associated with obesity (such as depression and anxiety) were reported to be more remarkable in females than males (Cooper et al., 2021) and the relationships between perceived stress and stress-related eating and weight gain were stronger in females when compared with males (Udo et al., 2014; Cotter and Kelly, 2018). Similarly, females showed baseline and final higher FSS and SCT time than age-matched males, suggesting the presence of a greater difficulty level in performing a common physical task, tentatively attributable to the gender-related lower (limb) muscle mass and strength, as already found in a previous study by our group (Rigamonti et al., 2019).

In contrast to our expectations, no correlation was found between ∆ % FSS (total score) and ∆ % SCT time, suggesting that a reduction of perceived fatigue was not directly accompanied by an improvement in muscle performance. The lack of correlation between the decrease in fatigue and the improvement of muscle performance might be due to the different measurements involved. In fact, while FSS is a self-report instrument that reflects a subjective perception of fatigue, SCT is an objective measure of muscle performance based on the measure of time spent doing a physical task (climb upstairs).

However, the improvement of SCT time in people who suffer from obesity reflects a functional improvement in performing the common actions of daily life, which are fundamental to increase the degree of autonomy of these individuals, amplified by the reduction in the subjective feeling of fatigue.

In addition, according to our results, such significant improvements are associated with a substantial improvement of the psychological state, as resulted in the PGWBI. In particular, levels of anxiety and depression decreased, while positive well-being, self-control, general health, and vitality significantly increased.

The above-reported positive results confirm and extend those obtained in previous works, where the same BWRP had been evaluated in adult and pediatric populations with obesity-related comorbidities (Rigamonti et al., 2020a,b). However, the novelty of the present study is having extended our knowledge on the beneficial effects of BWRP in elderly people with obesity and introduced a specific psychological measure capable to describe psychological well-being (PGWBI).

The current study presents some strengths that need to be pointed out, such as the relative large sample of elderly patients with a high rate of obesity, which made the sample of particular interest, the recruitment in a single clinical center delivering a high-quality in-hospital multidisciplinary BWRP, and the use of well-validated and widely-adopted objective and subjective measures (FSS, SCT; PGWBI).

As far as limitations of the study is concerned, the high specificity of the context where the study took place as well as the above-mentioned particularity of the sample (elderly people with a high rate of obesity) with an imbalance between males and females may limit the generalizability of the results and require caution before doing any extrapolation of the present results in a different context. However, we enrolled consecutively our population at the admission to the hospital, thus the study group actually reflects the natural composition of the adult obese populationafferent to our Institution (females approx. 2-fold more than males). In addition, the use of self-report measures of fatigue and psychological well-being should be limited by biases. Finally, the lack of follow-up measures prevents us to drive conclusions about the long-term effects of the intervention.

In conclusion, according to our results, BWRP provides encouraging results in terms of decreased BMI and fatigue, increased muscle performance, and enhanced psychological conditions in elderly people with obesity. Along with similar results obtained in previous studies, the present work provides additional support to the evidence that short-term BWRP entailing diet, physical activity, and psychological support is able to favorably modify and improve the physical and psychological conditions of elderly patients with obesity. This makes the BWRP capable to adhere to the recent recommendations by the most important scientific societies in clinical nutrition, geriatrics, and obesity (Villareal et al., 2005), according to which any BWRP administered in elderly obese patients, in addition to reduce obesity-associated medical complications, might focus on improving physical function and quality of life.

The novelty of this work is provided by the use of a varied pool of evaluation measures addressing both physical (BMI, FSS, SCT) and psychological outcomes (PGWBI) of a BWRP in a sample of particular interest from a clinical point of view, such as elderly patients with a high rate of obesity,

Further additional studies are required to address the main limitations of the current research (i.e., imbalance between males and females, possible confounding factors) and to evaluate the effective long-term maintenance of the positive effects with follow-up measures.

## Data availability statement

The raw data supporting the conclusions of this article will be made available by the authors, without undue reservation.

## Ethics statement

The studies involving human participants were reviewed and approved by Ethical Committee of Istituto Auxologico Italiano (registration code: 2013_06_27; project code: 18A301). The patients/participants provided their written informed consent to participate in this study.

## Author contributions

AG and AS designed the study. MB, AB, and DC helped to acquire the clinical data. AG analyzed the data and drafted this manuscript for the work. GC and AS reviewed the manuscript and provided final approval for the manuscript to be published. All authors read and approved the final manuscript.

## Funding

Research funded by the Italian Ministry of Health.

## Conflict of interest

The authors declare that the research was conducted in the absence of any commercial or financial relationships that could be construed as a potential conflict of interest.

## Publisher’s note

All claims expressed in this article are solely those of the authors and do not necessarily represent those of their affiliated organizations, or those of the publisher, the editors and the reviewers. Any product that may be evaluated in this article, or claim that may be made by its manufacturer, is not guaranteed or endorsed by the publisher.

## Supplementary material

The Supplementary material for this article can be found online at: https://www.frontiersin.org/articles/10.3389/fnagi.2022.1054941/full#supplementary-material
