# Supplementary material for: A three-week in-hospital multidisciplinary body weight reduction program exerts beneficial effects on physical and mental health and fatiguability of elderly patients with obesity
Source: Front Aging Neurosci. 2022 Dec 16;14:1054941. doi: 10.3389/fnagi.2022.1054941 (PMC9800933; doi:10.3389/fnagi.2022.1054941)

Supplementary Material

# Supplementary Figure

Figure 1. Correlations of ∆ % FSS (total score) and ∆ % SCT time (s).


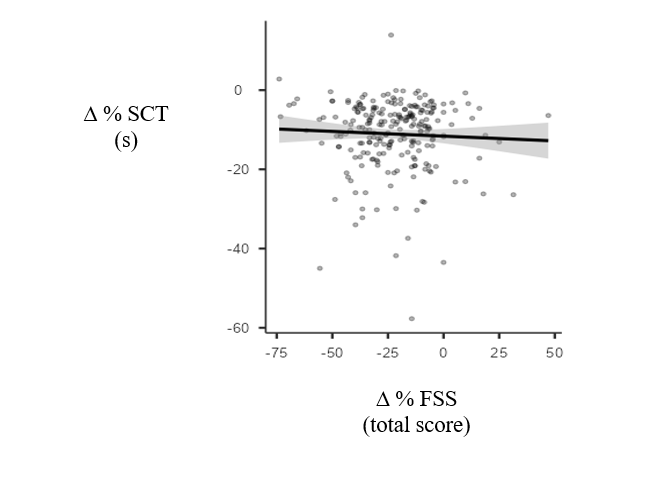

Supplement: Supplementary file 1 [file Data_Sheet_1.docx]
